# Mucosa-Associated Lymphoid Tissue (MALT) Lymphoma of the Urinary Bladder: A Case Report of a Rare Presentation

**DOI:** 10.7759/cureus.60885

**Published:** 2024-05-23

**Authors:** Susana Costa, Sérgio Chacim, Ângelo Oliveira, Carla Castro

**Affiliations:** 1 Radiation Oncology, Instituto Português de Oncologia do Porto, Porto, PRT; 2 Hematology, Instituto Português de Oncologia do Porto, Porto, PRT

**Keywords:** multimodal approach, rituximab, radiotherapy, malt lymphoma, bladder

## Abstract

Primary lymphoma of the urinary bladder is extremely rare. We present the case of a 67-year-old woman diagnosed with primary extranodal marginal zone lymphoma of mucosa-associated lymphoid tissue (MALT) of the urinary bladder. The patient presented with macroscopic hematuria. Renal ultrasound revealed a solid vascularized mass, in the inferior wall of the bladder. Pelvic computed tomography (CT) and magnetic resonance imaging (MRI) confirmed the presence of a polypoid lesion on the left side of the inferior bladder wall, measuring 40x45 mm, and the MRI study with gadolinium revealed that the entire bladder wall was involved. The patient underwent transurethral resection of the bladder tumor, demonstrating a histologic extensive involvement of bladder tissue by MALT lymphoma. The patient was treated with radiotherapy (24 Gy in 12 fractions) and four cycles of rituximab. She remained without evidence of disease 12 months later.

## Introduction

Marginal zone lymphoma (MZL) is an indolent disease and represents 7% of all non-Hodgkin lymphomas (NHLs) [1]. The World Health Organization recognizes three subtypes according to the primary site of involvement: extranodal MZL (EMZL) of mucosa-associated lymphoid tissue (MALT), nodal MZL, and splenic MZL [2]. MALT lymphoma occurs more commonly in the stomach (30%). It is less frequently observed in the orbit and ocular adnexa (12%), skin (10%), lung (9%), and salivary gland (7%) [1].

Primary malignant lymphoma of the urinary bladder is an extremely rare disease, corresponding to less than 0.2% of NHLs and less than 1% of bladder tumors [3]. Less than 70 cases have been described in the literature [3,4] since the first publication in 1990 [5]. Therefore, there is no consensus regarding its management. We report the clinical case of a woman with MALT lymphoma of the urinary bladder, describing the symptoms, diagnosis, treatment, and follow-up.

## Case presentation

**Figure 1:**
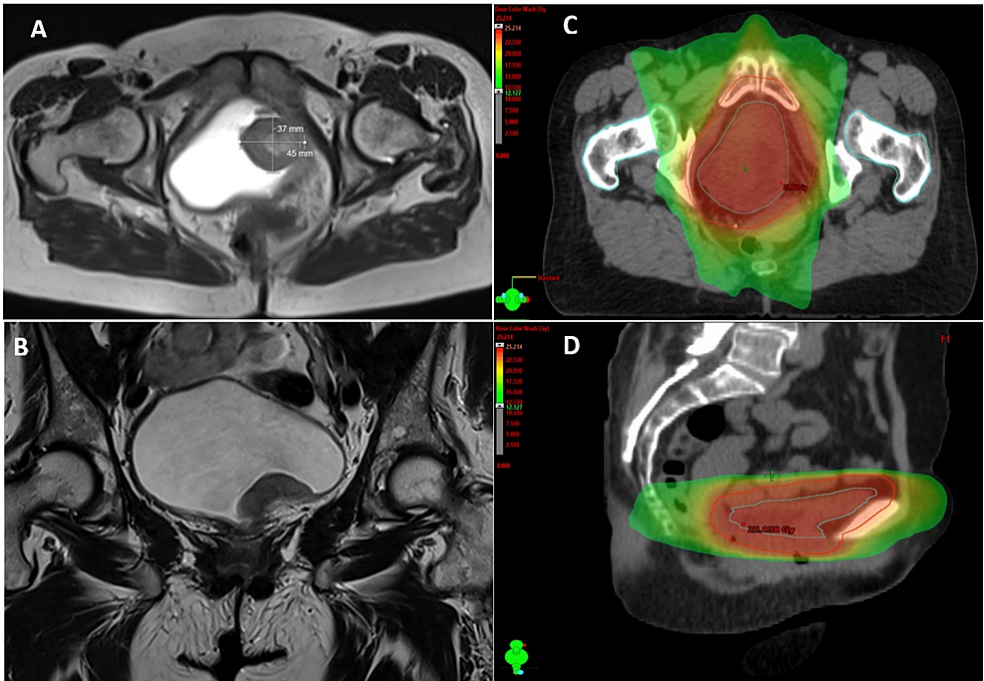
MALT lymphoma of the urinary bladder Axial (A) and coronal (B) planes of the pelvic MRI (T2-weighted) showing a mass invading the left side of the inferior urinary bladder wall, measuring 37x40 mm (APxT). Simulation CT and color-wash representation of the delineation of the clinical target volume (empty bladder; blue line) and planning tumor volume (red line) in axial (C) and sagittal (D) planes. MALT: mucosa-associated lymphoid tissue; MRI: magnetic resonance imaging; CT: computed tomography

This case presents a 67-year-old female patient, with a history of hypertension, depression, a bone cyst removed surgically, a femur fracture treated conservatively in 1988, and an appendectomy treated in 1983. There was no history of recurrent urinary tract infection. The patient presented with macroscopic hematuria, and a renal ultrasound was performed that showed a solid vascularized mass, in the inferior wall of the bladder, lateralized to the left. Chest, abdominal, and pelvis contrast-enhanced computed tomography (CT) and magnetic resonance imaging (MRI) (Figure 1) confirmed the presence of a polypoid lesion on the left side of the inferior bladder wall, measuring 37x40 mm (APxT), maximum thickness of 23 mm. MRI contrast revealed the involvement of the entire bladder wall. The lesion affected the bladder neck and the left uretero-vesical meatus, without apparent obstruction. No densification of adjacent fat or lymphadenopathy was observed. No pulmonary metastases were detected. Blood count and biochemistry were normal. Asymptomatic urinary infection caused by *Escherichia coli* was detected and treated with phosphomycin.

The patient underwent partial transurethral resection of the bladder mass. Microscopic examination demonstrated extensive involvement by lymphoid neoplasia with characteristics of B-cell lymphoma of the marginal zone, subtype MALT. Immunohistochemically, tumor cells showed expression of CD20 and no expression of CD3, CD5, CD10, CD21, CD23, Bcl6, or cyclin D1. Bone marrow biopsy was negative for malignant cells.

The case was discussed at a multidisciplinary meeting, and the patient was proposed for radical external radiotherapy followed by rituximab. Simulation CT was performed with an empty bladder, and the bladder was defined as the clinical target volume (CTV). Planning tumor volume (PTV) was defined as a 10-mm margin expansion from CTV, with 12 mm for the inferior margin. It was prescribed a total dose of 24 Gy in 12 fractions (BED_10_ 28.8 Gy), one fraction per day, daily, five days a week, in a total treatment time of 16 days. The treatment was performed in a linear accelerator, with photons of 6 MV, resorting to the intensity-modulated radiation therapy (IMRT) technique and daily kilovoltage (KV) intrafraction monitoring. After, the patient received four cycles of rituximab 375 mg/m^2^, one cycle per week. The treatments were well tolerated, with no adverse effects (Common Terminology Criteria for Adverse Events (CTCAE) grade 0). MRI and cystoscopy performed four and six months after the treatments showed no evidence of residual disease, maintaining no evidence of disease 12 months after. THe patient reported no side effects from treatments until now.

## Discussion

Primary lymphoma of the bladder is rare, corresponding to less than 1% of all bladder tumors and 0.2% of NHLs [3]. Primary bladder MALT lymphoma is the most common pathological type, followed by high-grade diffuse large B-cell type of lymphoma. It occurs more commonly in women (ratio 1:4.8) [4,6]. The age of patients at diagnosis ranges from 17 to 88 years old [4].

The precise pathologic mechanisms of primary bladder MALT lymphoma are not completely known. According to the literature, around 40% of patients with primary bladder lymphoma have a precedent of chronic cystitis, with *Escherichia coli* being the most common etiologic agent [7,8]. Morita et al. [6] reported a case of autoimmune interstitial cystitis associated with bladder MALT lymphoma. Our patient presented an asymptomatic urinary infection, with *Escherichia coli* as the infectious agent.

Primary MALT lymphoma of the urinary bladder has an indolent clinical course. Most of the patients present with gross hematuria, followed by pollakiuria, urgency, and dysuria. The differential diagnosis includes inflammatory lesions, bladder cancer, and infection.

Regarding imaging, the tumor has similarities with transitional carcinoma, but the infiltration seems to be more extensive. The MALT lymphoma tumor presents most commonly as a sessile mass and less frequently as multiple sessile masses or a polypoid mass [9]. Forty percent of the tumors originate in the lateral walls of the bladder, 22% in the inferior wall, 10% in the posterior wall, and less frequently as a diffuse thickness of the bladder wall. Then, there are no specific differential radiological findings, demanding a biopsy of the lesion to confirm the diagnosis by histology.

There is no consensus on the optimal treatment strategy for primary bladder MALT lymphoma, and it depends on the clinical behavior of the tumor and the performance status and life expectancy of the patient. The treatments available include antibiotics [7,8], surgery [10], radiotherapy [11,12], chemotherapy [13], immunotherapy [14], or a combined approach. Rituximab (anti-CD20 monoclonal antibody) has been used alone or in combination in the treatment of MALT lymphomas, with good response rates (55-73%) [14]. CHOP (cyclophosphamide, doxorubicin, vincristine, prednisolone) is one of the chemotherapy regimen options, with good results for both low- and high-grade primary bladder lymphomas, as monotherapy or combined [13]. Radiotherapy is frequently used, especially in localized low-grade tumors or as adjuvant treatment after resection. Goda et al. [12] reported a 10-year recurrence-free rate of 76% and an overall survival rate of 87% for patients treated with radiotherapy. The International Lymphoma Radiation Oncology Group (ILROG) suggests for indolent extranodal lymphomas a dose range of 20-30 Gy [15]. In the literature, the dose received by primary bladder MALT lymphoma ranged from 24 to 36 Gy [4,10-13]. Regarding treatment planning, ILROG recommends for pelvic extranodal lymphomas, such as the bladder, to delineate the whole organ as CTV and expand the volume to the PTV by 1 cm. Our patient was treated with external radiotherapy in a total dose of 24 Gy followed by four cycles of rituximab.

MALT lymphoma confined to the bladder appears to have a good prognosis and a lower tendency to disseminate to non-MALT lymphoid organs. We observed a complete response to treatment, remaining with no evidence of disease one year later.

## Conclusions

We describe a rare case of MALT lymphoma of the urinary bladder, treated with radiotherapy and rituximab. The patient showed a complete response, remaining with no evidence of the disease. The prognosis of primary bladder MALT lymphoma is good. Nonetheless, it is necessary to continue to publish information regarding clinicopathologic characteristics, treatment management, and prognosis of primary MALT lymphoma of the urinary bladder to better the knowledge of this disease.
